# Does changing healthcare use signal opportunities for earlier detection of cancer? A review of studies using information from electronic patient records

**DOI:** 10.1016/j.canep.2021.102072

**Published:** 2022-02

**Authors:** Becky White, Cristina Renzi, Meena Rafiq, Gary A. Abel, Henry Jensen, Georgios Lyratzopoulos

**Affiliations:** a ECHO (Epidemiology of Cancer Healthcare and Outcomes, Department of Behavioural Science and Health, Institute of Epidemiology and Health Care (IEHC)), University College London, Gower Street, London WC1E 6BT, UK; b University of Exeter Medical School, St Luke’s Campus, Magdalen Road, Exeter EX1 2LU, UK; c Research Unit for General Practice, Bartholins Allé 2, 8000 Aarhus C, Denmark

**Keywords:** Early detection of cancer[MeSH Term], Signs and symptoms[MeSH Term], Healthcare use, Health records, Early diagnosis

## Abstract

**Background:**

It has been proposed that changes in healthcare use before cancer diagnosis could signal opportunities for quicker detection, but systematic appreciation of such evidence is lacking. We reviewed studies examining pre-diagnostic changes in healthcare utilisation (e.g. rates of GP or hospital consultations, prescriptions or diagnostic tests) among patients subsequently diagnosed with cancer.

**Methods:**

We identified studies through Pubmed searches complemented by expert elicitation. We extracted information on the earliest time point when diagnosis could have been possible for at least some cancers, together with variation in the length of such ‘diagnostic windows’ by tumour and patient characteristics.

**Results:**

Across twenty-eight studies, changes in healthcare use were observable at least six months pre-diagnosis for many common cancers, and potentially even earlier for colorectal cancer, multiple myeloma and brain tumours. Early changes were also identified for brain and colon cancer sub-sites.

**Conclusion:**

Changing healthcare utilisation patterns before diagnosis indicate that future improvements in diagnostic technologies or services could help to shorten diagnostic intervals for cancer. There is greatest potential for quicker diagnosis for certain cancer types and patient groups, which can inform priorities for the development of decision support tools.

## Highlights

• Emerging research has found that healthcare use changes months before eventual diagnosis of cancer.
• This could identify which tumour or patient groups could be diagnosed sooner, and how much sooner.
• Our review confirms that patients’ healthcare use changes at least six months before diagnosis for many common cancers.
• Some studies suggested that patients with myeloma, brain tumours, or colorectal cancers could be diagnosed even sooner.

## 1 Introduction

Promptly diagnosing cancer in patients who present with new symptoms is crucial for improving survival [1], [2], [3], [4] and patient experience [5]. However, appropriately suspecting the diagnosis of cancer in these patients remains a challenge [6], [7], as many cancers present with non-specific symptoms associated with a range of possible diagnoses of different severity and prognosis. This makes prompt and accurate diagnosis difficult, leading to diagnostic delays. Information from electronic health records (EHRs) remains a rich resource for supporting the diagnostic process and targeting improvement efforts [8], [9].

**Fig. 1:**
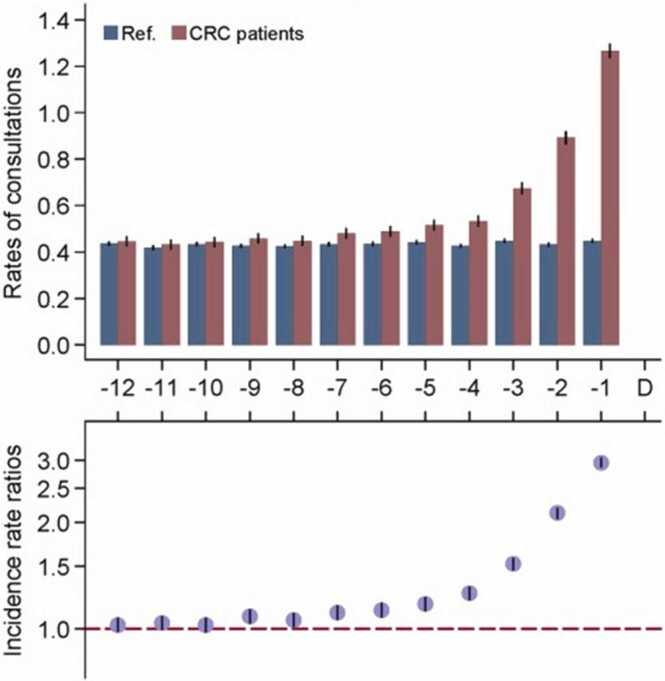
Exemplar evidence by Hansen et al examining healthcare utilisation changes before diagnosis of cancer. Illustrated for primary care consultations among women subsequently diagnosed with colorectal cancer, compared with controls. Reproduced with permission from John Wiley & Sons ©2015 UICC.

In cohorts of patients subsequently diagnosed with cancer, consultation rates, and the use of diagnostic tests or prescriptions are known to increase from baseline long before their diagnosis [10], [11], [12]. For example, rates of primary care consultations among women subsequently diagnosed with colorectal cancer started to increase from nine months before diagnosis, compared to controls (Fig. 1)[10]. The onset of changes in healthcare utilisation rates defines the start of a ‘diagnostic window’, during which quicker diagnosis would in principle be possible. This highlights opportunities to diagnose at least some of the patients sooner, by better appreciating and acting on the ‘signals’ indicated by changing patient healthcare utilisation [10], [13], or other signs and symptoms within the diagnostic window.

Nonetheless, there is currently no systematic appreciation of how much earlier cancer patients could be diagnosed in principle, as signalled by the onset of increasing healthcare use pre-diagnosis, and for which patients the potential opportunities are greatest. Further, the exact nature of different healthcare utilisation events that could be used to identify the onset of diagnostic windows is unclear. Motivated by these realisations, we reviewed evidence from population-based observational studies reporting on the patterns and timing of healthcare utilisation events before cancer diagnosis.

We aimed to summarise the maximum length of reported diagnostic windows, quantifying the earliest point that cancers can be diagnosed as indicated by changing patterns of consultations (and presenting signs and symptoms), prescriptions, diagnostic tests (and abnormal test results) or other changes in patterns of healthcare utilisation. We aimed to identify the earliest ‘inflection point’ identified by each study for each cancer type, defined as the point before diagnosis when rates of a pre-diagnostic event of interest increased above a background rate (or, as applicable to diagnostic tests, when average test values changed from a background rate). We also aimed to quantify any variation in the length of the diagnostic window by cancer type, as well as describing variation by other tumour and patient characteristics.

## 2 Methods

### 2.1 Search strategy and selection criteria

**Fig. 2:**
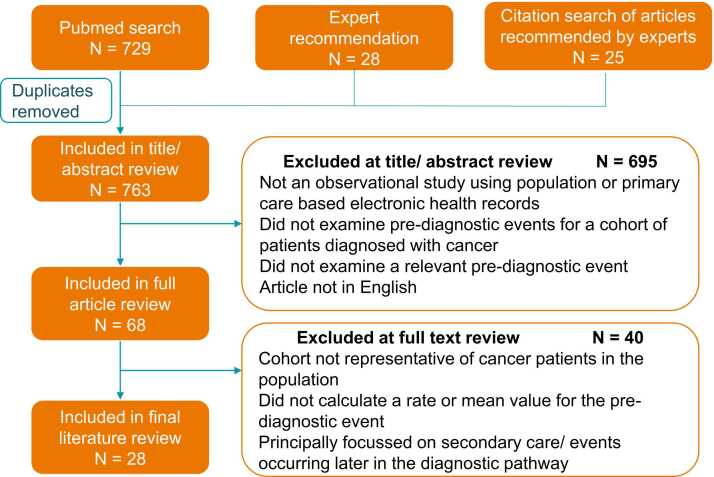
Flow diagram of numbers of studies identified and included in review.

Study selection followed a three-step process (Fig. 2). In step one, all studies published before 5th July 2021 were identified for inclusion through searches of the Pubmed database. The first search was for the key terms: cancer[Filter] AND early detection of cancer[MeSH Terms] AND (signs and symptoms[MeSH Terms] OR "before diagnosis" OR pre-diagnos* OR prediagnos* ). The second search used relevant author names identified via expert recommendation (see Appendix 1 for search terms). Additional studies were identified via expert recommendation by co-authors, and tracking citations within these recommended articles.

In step two, study titles or abstracts were inspected for whether they included patients diagnosed with cancer, and whether study populations related to patients registered to primary care, or all incident cases in the population. Included studies should have investigated the frequency and timing before diagnosis of one of the following event types: primary care consultations, secondary care consultations, medication prescriptions, diagnostic test use (and/or related test findings), or surgical procedures in relevant specialties. These event types were determined to be broadly relevant to the early detection of cancer by the authors of this study, based on clinical experience. There was no pre-defined list of specific relevant prescriptions, tests or surgical procedures of interest, as this would depend on the cancer type(s) examined by each study. Only articles available in English were included.

In step three, full articles were reviewed, if the cohort was representative of patients diagnosed with cancer in the population (e.g. excluding study cohorts from clinical trials, blood donor databases, or solely of patients with recurrent cancer). Studies principally focused on secondary care patients, or events that generally occur later in the diagnostic pathway or as part of confirming a malignancy (e.g. breast biopsy, breast mammogram) [14] were excluded. To identify changes over time, studies were included if they calculated a rate or mean value of a pre-diagnostic event using suitable time intervals (i.e. studies excluding the final year before diagnosis, or treating the entire pre-diagnostic period en bloc were deemed unsuitable and were excluded).

Studies should have explicitly reported an ‘inflection point’ in the article text. If a study did not do this, but included data in figures or tables that enabled its unambiguous identification, we extracted information about the first time period (e.g. month) when confidence intervals indicated that the outcome of interest (e.g. consultation rate) was significantly different to the time period immediately before (or where applicable, to controls).

A second author repeated the selection process for a random sub-group of 70 (9%) studies identified in step one, to check for concordance in study selection. The second author made the same decision (whether to include or exclude) for 98.6% (n = 69) of the studies, and the discordant study was excluded by consensus.

### 2.2 Summarising evidence on the length of the diagnostic window

We summarised the range of inflection points across the studies by event type, for all cancers combined and for specific cancer sites. Where studies reported more than one inflection point for the same event type (e.g. primary care consultations for relevant symptoms only and primary care consultations for any reason) or patient groups (e.g. males and females), the earliest single inflection point for the event type was chosen. The length of the diagnostic window was defined by the number of months between the extracted inflection point and cancer diagnosis. Finally, where reported, we extracted values for the diagnostic window length by tumour characteristics (e.g. tumour sub-site, presenting symptom), patient factors (e.g. sex, age), and other factors (e.g. route to diagnosis).

## 3 Results

### 3.1 Search yield and study selection

**Fig. 3:**
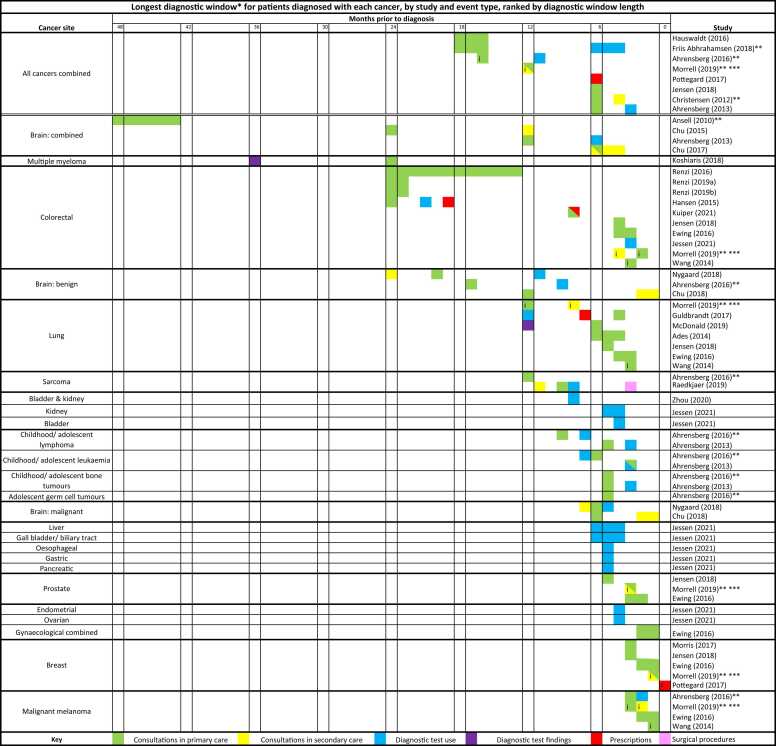
Longest diagnostic window* for patients diagnosed with each cancer, by study and event type, ranked by diagnostic window length. *The earliest point in time before diagnosis when a change was observed in a relevant clinical event type. Where multiple values were given by a study for an event type or patient groups, the earliest single value is shown. Therefore, the value shown may only apply to specific groups of patients with that cancer. For studies using longer/ shorter time intervals than months (e.g. quarters, days), the equivalent range of months are highlighted **Study included two different methods yielding different results; the results of primary focus in the study conclusions are shown here. ***Study examined 'GP' and 'specialist' consultations; these were assigned to primary and secondary care consultations, respectively. i Estimated by literature review authors using graphs or tables provided. ii No change before diagnosis.

763 studies were initially identified, of which 28 were included in the final review (Fig. 2) [10], [11], [12], [15], [16], [17], [18], [19], [20], [21], [22], [23], [24], [25], [26], [27], [28], [29], [30], [31], [32], [33], [34], [35], [36], [37], [38], [39]. All but four of the selected studies were carried out in Denmark or the UK, while the four remaining studies were set in Germany, Sweden, Australia, and the Netherlands. Selected studies were published between 2010 and 2021, and included patients diagnosed with any type of cancer, and/or 25 individual cancer sites (Fig. 3). Seven of the selected studies included children and young adults only, 17 included adults only, and four did not specify the age range.

### 3.2 Study designs

There was variation in methodological approaches, with 11 studies using a case-only and 17 a case-control study design. Identification of inflection point timing was either based on visual inspection or statistical estimation of the point when rates among cases changed either compared to baseline (in case-only studies), or corresponding synchronous rates among controls (in case-control studies) (Table 1). Four studies used more than one approach for inflection point identification, yielding different estimates within the same study [23], [28], [30], [31]. For two studies, we identified the inflection point using information on estimates and confidence intervals reported in the selected studies [25], [26]. In one of these, study authors identified some inflection points in the commentary which were different to those we identified using confidence intervals provided graphically [25]. Where the timing of the inflection point was compared between patient or tumour subgroups, no studies employed a formal statistical test.

**Table 1:** Summary of key methodological approaches used by published evidence to identify the onset of changing healthcare utilisation before cancer diagnosis (‘inflection points’), and recommendations for future research.

Methods used by studies* to identify inflection points	Considerations	Recommendations
1. Visual inspection of a time series graph to identify the time period when estimates among cases appeared to change (either compared to baseline for cases, or to controls) (13 studies)	Studies that identify the inflection point using statistical comparisons have better reproducibility than those using visual comparisons. However, some studies using statistical comparisons identified early changes that were statistically significant, though the observed variation in healthcare use between cases and controls was overall small (e.g. Hauswaldt et al.) [36].	Consider identifying the inflection point using statistical comparisons to improve reproducibility, bearing in mind that even small changes in rates of pre-diagnostic healthcare use may result in significant findings. Correction for type 1 errors caused by multiple testing may be needed (e.g. Bonferroni).
2. Statistical identification (case-only studies) of the first time period when estimates among cases were significantly different to a ‘baseline’ period (3 studies)	Where the inflection point is identified by comparing estimates in each time period to a ‘baseline’ period (as typically used in case-only studies), this appears to be sensitive to whether the inflection point is identified as the first time period that is statistically different to the period immediately before, or the start of observation period* . In the former approach, if changes are gradual, they may not be statistically different among adjacent periods (i.e. month by month). Moreover, in case-only studies, changes in healthcare use for cancer patients could reflect secular trends unrelated to cancer, changes in healthcare practice, or cohort ageing effects.	Where controls cannot be selected appropriately, case-only designs could be considered. However, consideration should be given to how the ‘baseline’ period is defined, as well as possible underlying secular trends, changing healthcare practice, and cohort ageing effects.
3. Statistical identification (case-control studies) of the first period when estimates among cases were significantly different to controls (13 studies)	In case-control designs, the background rate among controls can be used to account for underlying secular trends and other limitations of case-only study designs. However, the selection of appropriate controls can be challenging [40]. For example, a study of cases with lung cancer could not match controls on (or adjust the analysis for) smoking status (though adjustment for socioeconomic status likely minimised possible confounding by smoking status), thus potentially inflating the observed diagnostic window length [35].	The use of appropriately-designed case-control studies is encouraged to overcome limitations of case-only designs. However, simple comparisons between cases and controls in each time period could be sensitive to background differences between cases and controls, rather than pre-diagnostic changes in healthcare use among cases per se. Therefore, background estimates and secular trends in both cases and controls should be modelled.
4. Maximum likelihood estimation of the inflection point (i.e. identifying the time period for an inflection point which provides the best fit to the data) (1 study)	Comparison of model fit does not rely on there being statistically significant changes in estimates between individual time periods to identify an inflection point [15]. Moreover, underlying secular trends, changes in healthcare practice or cohort effects can be modelled.	This approach may circumvent issues in both case-only and case-control designs.

*For two studies, not shown here, we identified inflection points based on the estimates and confidence intervals provided. For Wang et al., we used method 2, identifying the first time period when estimates among cases were significantly different to the period immediately before [26]. We noted that results were different if comparing to the time period at the start of observation. For Morrell et al., we used method 3 [25]. Four studies used more than one approach for inflection point identification, yielding different estimates within the same study [23], [28], [30], [31].

There was variation in how the time before diagnosis was parameterised; weekly (N = 2), monthly (N = 18), bimonthly (N = 4), and longer time units (including quarters or six-month periods, and variable period lengths) (N = 7). Some studies used more than one time unit of analysis. Observation began at different points before diagnosis: 12 months (N = 8), 18 months (N = 3), two years (N = 9), three years (N = 3), four years (N = 1), and five years (N = 4). Overall study sample sizes ranged from 1606 [27] to 353,087 [39] patients. Further, for some studies using stratified analysis, the number of patients in specific groups was particularly low, for example under 100 patients [37].

### 3.3 Event types studied

Primary care consultations were the most widely studied event across cancer sites, examined by 25 studies, spanning 15 of the 16 individual cancer sites, and all cancers combined. Secondary care consultations were examined by seven studies, spanning nine cancer sites, and all cancers combined. There was heterogeneity between studies regarding the type of primary and secondary care consultations included. For example, regarding benign brain tumours, some studies included consultations for any symptom [11], [31], and some only included those for specific symptoms [20] (Appendix 2), with the specific symptoms considered further varying between studies. Heterogeneity also arose from whether primary care consultations via any contact method [20], or only face to face consultations were considered [11], [31]. This review did not compare diagnostic window length by contact method, as studies did not present findings by the specific method (e.g. face-to-face, email, telephone).

Diagnostic test use was examined by ten studies, spanning 20 individual cancer sites, and all cancers combined. Diagnostic test use encompassed imaging tests, biopsies, lung function tests, blood tests, urine tests, pulmonary function tests, electrocardiography, streptococcal throat infection, psychometric tests, and, in one study, unspecified ‘paraclinical’ examinations in particular hospital specialties [16]. Only two studies examined changes in diagnostic test findings, encompassing mean values for various blood tests, among patients subsequently diagnosed with multiple myeloma [12] and lung cancer [22]. Prescriptions were examined by four studies, spanning three individual cancer sites, plus all cancers combined. Surgical (i.e. orthopaedic, dermatology, and plastic surgery) procedures were only examined by a study of sarcoma patients [16].

### 3.4 Longest reported diagnostic windows by cancer site

For studies considering the outcome of all cancers combined, the length of the diagnostic window ranged from six [19], [23], [37], [39] to 16–18 months [28], [36] before diagnosis, as inferred from a detectable change in pre-diagnostic event rates or test values. The length of the diagnostic window varied substantially by cancer site (Fig. 3, Appendix 2).

The longest reported diagnostic windows (for all patients or at least a subgroup of patients) were four years (43–48 months) pre-diagnosis for all brain tumours combined [30], three years for multiple myeloma [12], and two years for colorectal cancer [10], [17], [18], [27], and benign brain tumours [24], [37]. Diagnostic windows of between six to 12 months were reported for lung cancer [22], [25], [35], sarcoma [16], [31], bladder and kidney cancers combined [15], childhood/ adolescent lymphoma and leukaemia [31], [37], malignant brain cancer [11], liver [34], and gall bladder/ biliary tract cancer [34].

Reported diagnostic windows were shortest (i.e. all under six months) for childhood/adolescent bone cancers [31], [37], adolescent germ cell tumours [31], oesophageal cancer [34], gastric cancer [34], pancreatic cancer [34], prostate cancer [19], [25], [38], breast cancer [19], [25], [32], [38], [39], malignant melanoma [25], [26], [31], [38], endometrial cancer [34], ovarian cancer [34], and gynaecological cancers combined [38].

### 3.5 Variation by tumour characteristics

Six studies examined variation by tumour characteristics. As indicated by changes in primary care consultation rates in two studies, reported diagnostic windows were generally longer for proximal colon compared to distal colon or rectal cancer [17], [33]. Increases in prescription rates for any newly-prescribed drug occurred earlier for proximal colon compared to distal colon or rectal cancer, [33] whereas increases in haemorrhoid prescription rates were earlier for rectal compared to colon cancers [10]. For brain tumours, window lengths varied by anatomic subsite (e.g. the supratentorial compartment, the midline, or cranial nerves) [21], and for some presenting symptoms (e.g. headache and convulsions), although patterns were complex [24]. For lung cancer, diagnostic windows did not vary by stage at diagnosis [29].

### 3.6 Variation by patient group

A study examining multiple cancer sites, and one studying all cancers combined found no differences in the length of the potential diagnostic window by sex [23], [26], while another including patients with brain cancer commented that differences existed, without specifying the pattern [11]. Five other studies did not comment specifically on differences in the diagnostic window length, but did stratify findings by sex [10], [12], [16], [18], [19], [31]. Some noted that sex stratification was needed given gender differences in baseline healthcare utilisation or comorbidities [10], [11], [18], [19]. Where examined, there was little evidence of variation in the inflection point by patients’ usual/ background consultation frequencies [19].

A study reported no differences in the length of the potential diagnostic window between patients diagnosed with colorectal cancer who were diagnosed either through an emergency presentation or through other diagnostic routes [27]. Two others examining colon cancer found likely differences in the diagnostic window length when considering comorbidity status and diagnostic route [17], [18]. For example, women with ‘serious’ non gastro-intestinal comorbidities who were diagnosed with colon cancer as an emergency had longer diagnostic windows, compared to non-comorbid women diagnosed either through emergency or non-emergency routes [18].

## 4 Discussion

### 4.1 Key findings

Evidence from electronic patient records indicates that for 15 common cancers, some patients begin to present at least six months before diagnosis. In the case of colorectal, brain tumours, and multiple myeloma, some studies suggest this may be even longer. The majority of this evidence was produced by studies examining increases in primary care consultations (including consultations for any reason or for specific presenting symptoms), but also included studies examining increases in secondary care consultations, diagnostic test use or changes in diagnostic test findings.

Longer diagnostic windows were identified for specific brain and colon cancer sub-sites, and for brain cancer patients, as indicated by increases in consultations for specific symptoms. Where studied, there was no evidence of, or limited variability in diagnostic window length by stage at diagnosis, sex, usual consultation frequency, or emergency presentation status (except for women with multi-morbidities diagnosed with colon cancer, and women diagnosed with proximal colon cancer) [17], [18], [19], [26], [27], [29].

### 4.2 Comparison with existing literature

We are not aware of previous reviews examining the length of potential diagnostic windows in patients with cancer. Some previous studies have estimated diagnostic intervals for individual patients, for example, from a presentation that is deemed a priori to be the first relevant one to the time of subsequently diagnosed cancer [41], [42], [43]. These studies rely on assumptions about how to define the ‘first relevant’ presentation, and achieving consistent definitions between studies is challenging, particularly in patients with morbidity who regularly consult for unrelated reasons [44]. The reviewed studies use a population approach in order to identify the earliest point at which healthcare utilisation rates change in some patients, avoiding the need for any such assumptions [20].

### 4.3 Limitations of the reviewed evidence

There are several limitations of the reviewed evidence. Firstly, evidence for 13 cancer sites (e.g. pancreatic cancer) was limited to single studies. It should be noted that for some of the cancer sites with the longest diagnostic windows (multiple myeloma and brain tumours), evidence of particularly long diagnostic windows of over one year was limited to one or two studies each. In addition, although the reviewed studies have the potential to illuminate disparities in the length of the diagnostic window between different patient groups, this has not yet been examined with regard to ethnicity, comorbidities, and age.

Studies used different methods to identify the onset of changes in healthcare utilisation (i.e. the timing of inflection points), potentially because they considered the measurement of diagnostic window length as a secondary or subsidiary aim. The exact timing of inflection points seems sensitive to the type of comparison used (i.e. whether through visual inspection or statistical approaches) and the study type used (i.e. case-only or case-control), as illustrated by studies that used more than one approach [23], [25], [28], [30], [31]. We have summarised and reflected on these methodological issues and related recommendations in Table 1.

In principle, the length of observed diagnostic windows may be influenced by the rate of tests performed, or the completeness of recording (e.g. of presenting symptoms). Regarding testing, greater or lower use of tests by doctors (e.g. as can be encountered in different study eras or different health systems) could impact the background rate of abnormal test results in either cases or controls in a population [12], [45]. If the background rate of testing is higher in cases, diagnostic windows may be longer in case-control studies. Regarding consultations, their occurrence is recorded reliably in electronic health record patient systems, so background rates should not differ systematically between cases and controls. However, the recording of a specific presenting symptom during a consultation could be mediated by the doctor’s perception of the patient’s risk of serious disease [46], [47]. Therefore, diagnostic windows related to rates of specific symptom presentations could be subject to similar biases to the recording of abnormal test results.

Power to detect inflection points is driven by the number of events in given time periods. Therefore, power may have been limited in certain studies, for example those using small samples of patients, short time units of analysis (e.g. monthly rather than quarterly rates), or examining relatively rare healthcare utilisation event types. However, shorter time units potentially offer more precise estimates of the timing of inflection points (for example, identifying the month, rather than the quarter where healthcare use begins to change from baseline). Limited power may also result from appropriate stratification by cancer site or gender.

All studies identifying differences in the inflection point between patient or tumour groups did so using stratified analyses, rather than formally testing for significant differences between groups. This approach does not account for potential confounding and has been shown to be open to misinterpretation [48]. Where relevant, differences in the inflection point between tumour or patient groups should be formally tested for statistical significance, for example by including the group as an interaction term in a model.

Finally, the length of the observation period before diagnosis varied by study. Longer observation periods to capture changing healthcare use should be recommended, as maximum reported diagnostic window lengths for some cancer sites are as long as two or three years.

### 4.4 Limitations of the review

It is possible that we did not identify some relevant papers, as some studies evaluated the diagnostic window to fulfil a subsidiary aim, so it may not have been mentioned in the abstract, title, or keywords. We therefore maximised coverage by including articles obtained via expert recommendation and searching the reference lists of articles already included.

Some of the observed variation between cancer sites in this review are likely explained by the aforementioned methodological variation between studies. Therefore, we have presented diagnostic windows by both cancer site and study in Fig. 3. As an illustration, in keeping with other case-only studies, we identified the inflection point in figures provided by Wang et al. as the first month when estimates among cases were different to the month before (their confidence intervals did not overlap) [26]. These figures would vary considerably if identified as the first month when estimates were different to the start of the observation period, however, this approach could be affected by gradual increases in healthcare utilisation as patients aged over the course of the study. Furthermore, due to stratification (e.g. by gender), the diagnostic window we extracted for some cancer sites may apply to a subset of patients, rather than to all patients diagnosed with that cancer (details are available in Appendix 2).

An additional source of variation between cancer sites was heterogeneity between studies in the healthcare events studied. Studies examined different healthcare events (e.g. consultations, prescriptions, tests), and also defined them in different ways. For example, some studies included all primary care consultations, while others only included those for specific symptoms, or via specific contact methods (e.g. face to face). Due to the other sources of variation between studies noted above (e.g. cancer site examined, study design), it was not possible in this review to quantify variation in diagnostic window length according to the type of healthcare event studied and how it was defined. A handful of studies explicitly examined and discussed this issue [10], [16], [23], [35], but further studies are needed, particularly those that include consultations for specific symptoms, and situated in healthcare systems other than Denmark. There is some evidence that diagnostic windows could vary according to the order in which healthcare events tend to occur in the patient’s diagnostic pathway. For example, a consultation with a GP tends to be the first event to occur, so studies examining this event may reveal earlier changes compared to those examining changes in diagnostic test use or abnormal test results [23].

### 4.5 Implications

The length of the diagnostic window after initial presentation to healthcare services could be influenced by tumour factors (e.g. cancer site, tumour aggressiveness, symptom signature), patient factors (e.g. comorbidities, patient engagement with healthcare services), and healthcare factors (e.g. type, timeliness, and availability of diagnostic investigations, and monitoring (‘safety-netting’) protocols). Longer diagnostic windows could indicate opportunities to diagnose cancer sooner in some patients. These could arise in patients with cancers characterised by early onset but non-specific symptoms, which are often either not immediately investigated, or investigated with non-specific tests that lead to complex and prolonged diagnostic pathways to eventual diagnosis. However, the exact mechanisms leading to potentially avoidable delays have not been established in the reviewed literature. Further research is therefore needed to help targeting of interventions to support the diagnostic process.

Although the literature suggests that time to diagnosis could be shortened in some patients, it may not necessarily reduce the proportion of patients diagnosed at an advanced stage of disease, because slowly progressing tumours may be over-represented among patients who experience long diagnostic intervals [4], [29], [49]. In addition, by its nature, the onset of a diagnostic window identified from a population will reflect the group of patients with the longest intervals between first presentation and diagnosis, with most patients having shorter diagnostic intervals. A more detailed understanding is needed regarding the proportion of patients whose diagnosis could be expedited, and by how long.

The findings indicate that there is potential to harness electronic health records to inform the management of patients in practice. Electronic health records could be used to develop diagnostic “e-triggers”; these could flag patients in whom the suspicion of cancer may require monitoring or repeat assessment [12], [15], [20], [35], for example, if patients consult more frequently than usual [19], [27], or receive particular prescriptions e.g. for haemorrhoids [10], which could raise suspicion of particular cancers. This prospect is particularly promising for patients in contact with healthcare services who are at increased underlying cancer risk (for example, due to their age or pre-existing comorbidities), who do not present with any specific ‘alarm’ symptoms for cancer that would usually qualify them for urgent referral [50].

In practice, identifying increased consultation frequency in individuals in a timely manner may be challenging, because most patients do not consult regularly at baseline [19], [36]. Where an increase in healthcare use is identified for a patient, the predictive value of increased consultation frequency is still likely to be low if considered in isolation, as consultations are relatively common events in the general population, compared to cancer. Therefore, an observed change in a patient’s individual consultation frequency may need to be combined with other clinical features (e.g. by presenting symptom, or history of additional diagnostic investigations) to better inform risk quantification [36].

## 5 Conclusion

Evidence of changing healthcare utilisation before cancer diagnosis recorded in electronic health records can be used to identify tumour or patient groups in which faster diagnosis could be achievable, and how much faster might be possible. With future improvements to the diagnostic process and diagnostic technologies, some patients could potentially be diagnosed with cancer at least six months earlier. Some studies suggested that for brain tumours, colorectal cancer, and multiple myeloma, some diagnoses could be made even sooner. Future research should seek to confirm this, and explore variation by tumour and patient groups, and additional cancer sites. Further consideration is needed about whether such clinical information could be harnessed in practice to improve the diagnostic process. Methodological considerations identified in this review can help to improve the design and consistency in reporting of future primary studies using electronic health records in this emerging field.

## Funding

This study was supported by the Cancer Research UK Fellowship award (C18081/A18180, to GL) (GL, BW), and is affiliated to the multi-institutional CanTest Research Collaborative funded by a Cancer Research UK Population Research Catalyst Award (C8640/A23385), of which GL is Associate Director, GAA Senior Faculty and CR, MR and BW Faculty members. The study aligns to (although is not directly supported by) the RREDD-HER project supported by the International Alliance for Cancer Early Detection (C18081/A31373).

## CRediT authorship contribution statement

BW, GL, and CR conceived and designed the study and agreed the search and data extraction strategy. BW identified and analysed the studies, under the supervision of GL and CR and with a sample of studies independently reviewed by CR. MR, GL and CR provided clinical input into interpretations. GA and HJ provided statistical and methodological expertise. All authors contributed to draughting and revising the article.

## Conflict of interest statement

The authors declare no competing interests.
