# Supplementary material for: Does changing healthcare use signal opportunities for earlier detection of cancer? A review of studies using information from electronic patient records
Source: Cancer Epidemiol. 2022 Feb;76:102072. doi: 10.1016/j.canep.2021.102072 (PMC8785122; doi:10.1016/j.canep.2021.102072)
Supplement: Supplementary file 1 — Supplementary material. [file mmc1.docx]

**Appendix 1. Pubmed search terms for author search**

cancer[Filter] AND (early detection of cancer[MeSH Terms] OR signs and symptoms[MeSH Terms] OR "before diagnosis" OR pre-diagnos* OR prediagnos*) AND (Ahrensberg JM[Author] OR Christensen KG [Author] OR Chu TPC[Author] OR "Ewing M"[Author] OR Friis Abrahamsen C[Author] OR Guldbrandt LM[Author] OR Hansen PL[Author] OR Hauswaldt J[Author] OR Koshiaris C[Author] OR "Jensen H"[Author] OR "McDonald L"[Author] OR Morrell S[Author] OR Nygaard C[Author] OR Pottegard A[Author] OR Raedkjaer M[Author] OR Renzi C[Author] OR "Wang Yingying"[Author] OR "Zhou Yin"[Author] OR "Nanna Holt Jessen"[Author] OR "Kuiper JG"[Author])
