# Supplementary material for: Does changing healthcare use signal opportunities for earlier detection of cancer? A review of studies using information from electronic patient records
Source: Cancer Epidemiol. 2022 Feb;76:102072. doi: 10.1016/j.canep.2021.102072 (PMC8785122; doi:10.1016/j.canep.2021.102072)
Supplement: Supplementary file 2 — Supplementary material. [file mmc2.docx]

**Appendix 2. Study settings, cohorts, and longest maximum diagnostic window for patients diagnosed with each cancer, by study and event type**

Cancers are ranked by maximum diagnostic window length, and within each cancer, studies are ranked by maximum diagnostic window length

| **Table 1. Study settings, cohorts, and longest maximum diagnostic window* for patients diagnosed with each cancer, by study and event type, ranked by diagnostic window length** | | | |
| --- | --- | --- | --- |
| Study | Setting & cohort (country: context; diagnosis dates; age range (N)) | Methods summary (design; method to identify inflection point; observation period pre-diagnosis) | Longest maximum diagnostic window for a single patient group, for each event type studied |
| **All cancers combined** | | | |
| Hauswaldt (2016) | Germany: primary care; cancers diagnosed 1996-2006 and with 1+ primary care contact < 18 months pre-diagnosis; age range not stated (N=3,310) | Statistical identification (case-control); earliest quarter when the inter-contact interval (time lag between two consecutive consultations) was shorter among cases than controls; < 18 months pre-diagnosis | 16-18 months (decrease in interval between primary care consultations for any reason) |
| Friis Abhrahamsen (2018) | Denmark: primary care; cancers diagnosed 2008-2015; children aged < 15 years (N=1,386) | Statistical identification (case-control); earliest quarter when rates were significantly higher among cases than controls; < 24 months pre-diagnosis** | 16-18 months (increase in primary care consultations for any reason) 4-6 months (increase in primary care diagnostic test use (urine tests, blood tests, pulmonary function, electrocardiography, streptococcal throat infection)) |
| Ahrensberg (2016) | Denmark: primary care; cancers diagnosed 2002-2011; young adults 15-39 years (N=12,306) | Statistical identification (case-control); earliest month when rates were significantly higher among cases than controls (and increase was sustained); < 24 months pre-diagnosis** | 16 months (increase in primary care consultations for any reason)** 11 months (increase in primary care blood test use) |
| Morrell (2019) | New South Wales, Australia: primary and secondary care; cancers diagnosed 2006-2015; adults aged 45 years + (N=16,750) | Statistical identification (case-control); earliest month when proportions were significantly higher among cases than controls; < 12 months pre-diagnosis** | 12 months (increase in GP consultations for any reason) 12 months (increase in specialist consultations for any reason, emergency day visits and emergency inpatient admissions) |
| Pottegard (2017) | Denmark: primary care; cancers diagnosed 2000-2012; adults (N=353,087) | Visual identification (case-control); earliest month when rates among cases appeared to increase; < 24 months pre-diagnosis | 6 months (increase in new first-time prescription use for any drug) |
| Jensen (2018) | Denmark: primary care; cancers diagnosed 2009-2013; adults aged 50-90 years (N=123,943) | Statistical identification (case-only); earliest month when rates were significantly higher among cases compared to cases in the preceding month; < 18 months pre-diagnosis | 6 months (increase in primary care consultations for any reason for males) |
| Christensen (2012) | Denmark:primary and secondary care; cancers diagnosed 2001-2006; adults aged 40 years + (N=127,210) | Visual identification (case-control); earliest month when rates among cases appeared to increase/ earliest quarter when rates were significantly higher among cases than controls; < 12 months pre-diagnosis** | 6 months (increase in primary care consultations for any reason) 4 months (increase in secondary care admissions and outpatient visits for any reason) 4 months (increase in diagnostic test use (x ray, ultrasound, endoscopy, biopsies, CAT scan, MRI scan, angiography) within particular specialties)) |
| Ahrensberg (2013) | Denmark: primary care; cancers diagnosed 2002-2008; children < 16 years (N=1,278) | Statistical identification (case-control); earliest month when rates were significantly higher among cases than controls; < 12 months pre-diagnosis | 6 months (increase in primary care daytime consultations for any reason) 3 months (increase in primary care diagnostic test use) |
| **Brain: benign & malignant combined** | | | |
| Ansell (2010) | UK: primary care; cancers diagnosed 1992-1996 ; children 1-14 years (N=195) | Statistical identification (case-control); earliest 6-month interval when rates were significantly higher among cases than controls; < 4 years pre-diagnosis** | 4 years (43-48 months) (increase in primary care consultations (relevant symptoms only), increase in records of relevant symptoms in primary care) |
| Chu (2015) | England; primary and secondary care; cancers diagnosed 1989-2006; children & young adults (N=9,799) | Visual identification (case-only); earliest month when smoothed rates among cases appeared to increase; < 36 months pre-diagnosis | 24 months (increase in primary care consultations for headache and growth/ endocrine disorders) 12 months (increase in secondary care consultations for convulsions) |
| Ahrensberg (2013) | Denmark: primary care; cancers diagnosed 2002-2008; children < 16 years (N=298) | Statistical identification (case-control); earliest month when rates were significantly higher among cases than controls; < 12 months pre-diagnosis | 12 months (increase in primary care daytime consultations for any reason) 6 months (increase in primary care diagnostic test use) |
| Chu (2017) | England; primary and secondary care; cancers diagnosed 1989-2006; children & young adults (N=9,799) | Visual identification (case-only); earliest month when smoothed rates among cases appeared to increase; < 36 months pre-diagnosis | 6 months (increase in primary care consultations for relevant symptoms only) 3-6 months (increase in secondary care consultations for relevant symptoms only) |
| **Multiple myeloma** | | | |
| Koshiaris (2018) | England: primary care; cancers diagnosed 2000-2009; adults > 40 years old (N=2,703) | Statistical identification (case-control); earliest quarter when rates were significantly higher/ mean test values were significantly different among cases compared to controls; < 5 years pre-diagnosis | 24 months (increase in primary care consultations for specific symptom groups (back pain, rib pain, chest infections, chest pain, nosebleed)) 36 months (decrease in primary care mean haemoglobin values) |
| **Colorectal** | | | |
| Renzi (2016) | England: primary care; cancers diagnosed 2005-2006; adults 25 years + (N=1,606) | Statistical identification (case-only); earliest time interval (time interval size varied) when rates were significantly higher among cases compared to cases in the preceding time interval; < 5 years pre-diagnosis | 13-24 months (increase in primary care consultations for any reason) |
| Renzi (2019a) | England: primary care; cancers diagnosed 2005-2010; adults 18 years + (colon only, N=5,745) | Visual identification (case-only); earliest two-month interval when rates among cases appeared to increase; < 5 years pre-diagnosis | 23-24 months (increase in primary care consultations for relevant symptoms for female emergency presenters with 'serious' non gastro-intestinal comorbidities diagnosed/ treated in secondary care) |
| Renzi (2019b) | England: primary care; cancers diagnosed 2005-2010; adults > 18 years (colon only, N=5,745) | Visual identification (case-only); earliest two-month interval when rates among cases appeared to increase; < 5 years pre-diagnosis | 23-24 months (increase in primary care consultations for relevant symptoms for females with proximal colon cancer diagnosed as an emergency) |
| Hansen (2015) | Denmark: primary care; cancers diagnosed 2004-2010; adults aged 40-80 years (N=19,209) | Statistical identification (case-control); earliest month when rates were significantly higher among cases than controls; < 12 months pre-diagnosis (extended to 24 months for some events) | 24 months (increase in primary care consultations for any reason for females with proximal colon cancer) 21 months (increase in primary care haemoglobin test use for males with proximal colon cancer) 19 months (increase in primary care haemorrhoid prescription use for females with rectal cancer) |
| Kuiper (2021) | Netherlands: primary care; cancers diagnosed 2007-2014; age range not stated (N=6,087) | Statistical identification (case-control); earliest month when rates were significantly higher among cases than controls; < 12 months pre-diagnosis | 8 months (increase in primary care consultations for any reason for patients with proximal colon cancer) 8 months (increase in prescriptions for any drug for patients with proximal colon cancer) |
| Jensen (2018) | Denmark: primary care; cancers diagnosed 2009-2013; adults aged 50-90 years (N=17,138) | Statistical identification (case-only); earliest month when rates were significantly higher among cases compared to cases in the preceding month; < 18 months pre-diagnosis | 4 months (increase in primary care consultations for any reason for males who usually consult 'rarely') |
| Ewing (2016) | Sweden: primary care; cancers diagnosed 2011 and with 1+ primary care contact < 12 months pre-diagnosis; adults (N=753) | Visual identification (case-control); earliest week (reported as days) when rates among cases appeared to increase; < 12 months pre-diagnosis | 3-4 months (100 days) (increase in primary care consultations for any reason, increase in records of diagnostic codes in primary care) |
| Jessen (2021) | Denmark: primary and secondary care; cancers diagnosed 2014-2018; age range not stated (colon N=15,017, rectal N=7,176) | Statistical identification (case-only); earliest month when rates were significantly higher among cases compared to cases in the preceding month; < 12 months pre-diagnosis | 3 months (increase in colonoscopy use for patients with colon or rectal cancer) |
| Morrell (2019) | New South Wales, Australia: primary and secondary care; cancers diagnosed 2006-2015; adults aged 45 years + (N=2,077) | Statistical identification (case-control); earliest month when proportions were significantly higher among cases than controls; < 12 months pre-diagnosis** | 2 months (increase in GP consultations for any reason)** 4 months (increase in emergency inpatient admissions)** |
| Wang (2014) | UK; primary care; cancers diagnosed 1997-2006; adults (N=12,189) | Statistical identification (case-only); earliest month when rates were significantly higher among cases compared to cases in the preceding month (identified by literature review authors); < 24 months pre-diagnosis | 3 months (increase in primary care consultations for any reason for males)** |
| **Brain: benign** | | | |
| Nygaard (2018) | Denmark; primary and secondary care; cancers diagnosed 2009-2014; adults (N=3,654) | Statistical identification (case-control); earliest month when rates were significantly higher among cases than controls; < 24 months pre-diagnosis | 20 months (increase in primary care consultations for any reason, for females) 24 months (increase in secondary care consultations in Ear-Nose-Throat speciality for males/ females, all other hospital contacts for males) 11 months (increase in radiology test use for males) |
| Ahrensberg (2016) | Denmark: primary care; cancers diagnosed 2002-2011; young adults 15-39 years N=1,569) | Statistical identification (case-control); earliest month when rates were significantly higher among cases than controls (and increase was sustained); < 24 months pre-diagnosis** | 17 months (increase in primary care consultations for any reason) 9 months (increase in primary care blood test use) 6 months (increase in primary care psychometric test use) |
| Chu (2018) | England; primary and secondary care; cancers diagnosed 1989-2006; children & young adults (N=9,799)**** | Visual identification (case-only); earliest month when smoothed rates among cases appeared to increase; < 36 months pre-diagnosis | 12 months (increase in primary care consultations for relevant symptoms only) 1-2 months (increase in secondary care consultations for relevant symptoms only) |
| **Lung** | | | |
| Morrell (2019) | New South Wales, Australia: primary and secondary care; cancers diagnosed 2006-2015; adults aged 45 years + (N=1,235) | Statistical identification (case-control); earliest month when proportions were significantly higher among cases than controls; < 12 months pre-diagnosis** | 12 months (increase in GP consultations for any reason)** 8 months (increase in specialist consultations for any reason)** |
| Guldbrandt (2017) | Denmark: primary care; cancers diagnosed 2003-2012; adults aged 40-90 years (N=34,017) | Statistical identification (case-control); earliest month when rates were significantly higher among cases than controls; < 12 months pre-diagnosis | 4 months (increase in primary care consultations for any reason) 12 months (increase in first-time primary care lung function test use) 12 months (increase in first-time radiology test use) 7 months (increase in new COPD prescription use) |
| McDonald (2019) | UK: primary care; cancers diagnosed 1887-2018; adults (N=26,379) | Statistical identification (case-control); earliest two-month interval when rates/ proportions were significantly higher among cases than controls; < 24 months pre-diagnosis | 6 months (increase in records of relevant symptoms in primary care) 12 months (increase in proportion of patients in primary care with high CRP test values) |
| Ades (2014) | Devon, UK: primary care; cancers diagnosed 1998-2002; age range not stated (N=247) | Visual identification (case-control); earliest quarter when rates among cases appeared to increase; < 24 months pre-diagnosis | 4-6 months (increase in records of two relevant symptoms per quarter in primary care) |
| Jensen (2018) | Denmark: primary care; cancers diagnosed 2009-2013; adults aged 50-90 years (N=17,861) | Statistical identification (case-only); earliest month when rates were significantly higher among cases compared to cases in the preceding month; < 18 months pre-diagnosis | 5 months (increase in primary care consultations for any reason for females who usually consult with 'average' frequency) |
| Ewing (2016) | Sweden: primary care; cancers diagnosed 2011 and with 1+ primary care contact < 12 months pre-diagnosis; adults (N=373) | Visual identification (case-control); earliest week (reported as days) when rates among cases appeared to increase; < 12 months pre-diagnosis | 3-4 months (100 days) (increase in primary care consultations for any reason, increase in records of diagnostic codes in primary care) |
| Wang (2014) | UK; primary care; cancers diagnosed 1997-2006; adults (N= 11,081) | Statistical identification (case-only); earliest month when rates were significantly higher among cases compared to cases in the preceding month (identified by literature review authors); < 24 months pre-diagnosis | 3 months (increase in primary care consultations for any reason for males/ females)** |
| **Sarcoma** | | | |
| Ahrensberg (2016) | Denmark: primary care; cancers diagnosed 2002-2011; young adults 15-39 years (soft tissue sarcoma only N=315) | Statistical identification (case-control); earliest month when rates were significantly higher among cases than controls (and increase was sustained); < 24 months pre-diagnosis** | 12 months (increase in primary care consultations for any reason) |
| Raedkjaer (2019) | Denmark: primary and secondary care; cancers diagnosed 2000-2013; adults (N=2,167) | Statistical identification (case-control); earliest month when rates were significantly higher among cases than controls; < 24 months pre-diagnosis | 9 months (increase in primary care consultations for any reason) 11 months (increase in secondary care inpatient consultations for any reason, within orthopaedic surgery, dermatology, plastic surgery) 3 months (increase in secondary care surgery, within orthopaedic surgery, dermatology, plastic surgery) 8 months (increase in secondary care paraclinical examinations) |
| **Bladder & kidney combined** | | | |
| Zhou (2020) | England; primary and secondary care; cancers diagnosed 2012-2015; adults 25 years+ (N=2,971) | Statistical identification through model comparison (case-only); models of monthly rates were fitted with different likely inflection points, with the model with optimal goodness of fit chosen; < 12 months pre-diagnosis | 8 months (increase in x-ray use) |
| **Kidney** | | | |
| Jessen (2021) | Denmark: primary and secondary care; cancers diagnosed 2014-2018; age range not stated (N=4,224) | Statistical identification (case-only); earliest month when rates were significantly higher among cases compared to cases in the preceding month; < 12 months pre-diagnosis | 4-5 months (increase in abdominal CT use) |
| **Bladder** | | | |
| Jessen (2021) | Denmark: primary and secondary care; cancers diagnosed 2014-2018; age range not stated (N=3,801) | Statistical identification (case-only); earliest month when rates were significantly higher among cases compared to cases in the preceding month; < 12 months pre-diagnosis | 4 months (increase in transvaginal ultrasound use) |
| **Childhood/ adolescent lymphoma** | | | |
| Ahrensberg (2016) | Denmark: primary care; cancers diagnosed 2002-2011; young adults 15-39 years (N=765) | Statistical identification (case-control); earliest month when rates were significantly higher among cases than controls (and increase was sustained); < 24 months pre-diagnosis** | 9 months (increase in primary care consultations for any reason) 7 months (increase in primary care blood test use for leukaemia & lymphoma combined) |
| Ahrensberg (2013) | Denmark: primary care; cancers diagnosed 2002-2008; children < 16 years (N=105) | Statistical identification (case-control); earliest month when rates were significantly higher among cases than controls; < 12 months pre-diagnosis | 5 months (increase in primary care daytime consultations for any reason) 3 months (increase in use of primary care diagnostic tests) |
| **Childhood/ adolescent leukaemia** | | | |
| Ahrensberg (2016) | Denmark: primary care; cancers diagnosed 2002-2011; young adults 15-39 years (N=386) | Statistical identification (case-control); earliest month when rates were significantly higher among cases than controls (and increase was sustained); < 24 months pre-diagnosis** | 6 months (increase in primary care consultations for any reason) 7 months (increase in primary care blood test use for leukaemia & lymphoma combined) |
| Ahrensberg (2013) | Denmark: primary care; cancers diagnosed 2002-2008; children < 16 years (N=354) | Statistical identification (case-control); earliest month when rates were significantly higher among cases than controls; < 12 months pre-diagnosis | 3 months (increase in primary care daytime consultations for any reason) 3 months (increase in use of primary care diagnostic tests) |
| **Childhood/ adolescent bone tumours** | | | |
| Ahrensberg (2016) | Denmark: primary care; cancers diagnosed 2002-2011; young adults 15-39 years (N=144) | Statistical identification (case-control); earliest month when rates were significantly higher among cases than controls (and increase was sustained); < 24 months pre-diagnosis** | 5 months (increase in primary care consultations for any reason) |
| Ahrensberg (2013) | Denmark: primary care; cancers diagnosed 2002-2008; children < 16 years (N=65) | Statistical identification (case-control); earliest month when rates were significantly higher among cases than controls; < 12 months pre-diagnosis | 5 months (increase in primary care daytime consultations for any reason) 3 months (increase in use of primary care diagnostic tests) |
| **Adolescent germ cell tumours** | | | |
| Ahrensberg (2016) | Denmark: primary care; cancers diagnosed 2002-2011; young adults 15-39 years (N=1,837) | Statistical identification (case-control); earliest month when rates were significantly higher among cases than controls (and increase was sustained); < 24 months pre-diagnosis** | 5 months (increase in primary care consultations for any reason) |
| **Brain: malignant** | | | |
| Nygaard (2018) | Denmark; primary and secondary care; cancers diagnosed 2009-2014; adults (N=2,272) | Statistical identification (case-control); earliest month when rates were significantly higher among cases than controls; < 24 months pre-diagnosis | 6 months (increase in primary care consultations for any reason for males) 7 months (increase in secondary care consultations in neurology, for females) 5 months (increase in radiology test use for males/ females) |
| Chu (2018) | England; primary and secondary care; cancers diagnosed 1989-2006; children & young adults (N=9,799)**** | Visual identification (case-only); earliest month when smoothed rates among cases appeared to increase; < 36 months pre-diagnosis | 6 months (increase in primary care consultations for relevant symptoms only) 1-2 months (increase in secondary care consultations for relevant symptoms only) |
| **Liver** | | | |
| Jessen (2021) | Denmark: primary and secondary care; cancers diagnosed 2014-2018; age range not stated (N= 2,028) | Statistical identification (case-only); earliest month when rates were significantly higher among cases compared to cases in the preceding month; < 12 months pre-diagnosis | 4-6 months (increase in abdominal ultrasound use) |
| **Gall bladder/ biliary tract** | | | |
| Jessen (2021) | Denmark: primary and secondary care; cancers diagnosed 2014-2018; age range not stated (N=906) | Statistical identification (case-only); earliest month when rates were significantly higher among cases compared to cases in the preceding month; < 12 months pre-diagnosis | 4-6 months (increase in abdominal ultrasound use) |
| **Oesophageal** | | | |
| Jessen (2021) | Denmark: primary and secondary care; cancers diagnosed 2014-2018; age range not stated (N= 2,263) | Statistical identification (case-only); earliest month when rates were significantly higher among cases compared to cases in the preceding month; < 12 months pre-diagnosis | 5 months (increase in gastroscopy use) |
| **Gastric** | | | |
| Jessen (2021) | Denmark: primary and secondary care; cancers diagnosed 2014-2018; age range not stated (N=2,660) | Statistical identification (case-only); earliest month when rates were significantly higher among cases compared to cases in the preceding month; < 12 months pre-diagnosis | 5 months (increase in gastroscopy use) |
| **Pancreatic** | | | |
| Jessen (2021) | Denmark: primary and secondary care; cancers diagnosed 2014-2018; age range not stated (N=4,304) | Statistical identification (case-only); earliest month when rates were significantly higher among cases compared to cases in the preceding month; < 12 months pre-diagnosis | 5 months (increase in gastroscopy or endoscopic retrograde cholangiopancreatography use) |
| **Prostate** | | | |
| Jensen (2018) | Denmark: primary care; cancers diagnosed 2009-2013; adults aged 50-90 years (N=19,348) | Statistical identification (case-only); earliest month when rates were significantly higher among cases compared to cases in the preceding month; < 18 months pre-diagnosis | 5 months (increase in primary care consultations for any reason for males who usually consult with 'average' frequency) |
| Morrell (2019) | New South Wales, Australia: primary and secondary care; cancers diagnosed 2006-2015; adults aged 45 years + (N=3,960) | Statistical identification (case-control); earliest month when proportions were significantly higher among cases than controls; < 12 months pre-diagnosis** | 3 months (increase in GP consultations for any reason)** 3 months (increase in specialist consultations for any reason and emergency day visits)** |
| Ewing (2016) | Sweden: primary care; cancers diagnosed 2011 and with 1+ primary care contact < 12 months pre-diagnosis; adults (N=1,257) | Visual identification (case-control); earliest week (reported as days) when rates among cases appeared to increase; < 12 months pre-diagnosis | 2-3 months (80 days) (increase in primary care consultations for any reason, increase in records of diagnostic codes in primary care) |
| **Endometrial** | | | |
| Jessen (2021) | Denmark: primary and secondary care; cancers diagnosed 2014-2018; age range not stated (N=3,517) | Statistical identification (case-only); earliest month when rates were significantly higher among cases compared to cases in the preceding month; < 12 months pre-diagnosis | 4 months (increase in transvaginal ultrasound use) |
| **Ovarian** | | | |
| Jessen (2021) | Denmark: primary and secondary care; cancers diagnosed 2014-2018; age range not stated (N=2,002) | Statistical identification (case-only); earliest month when rates were significantly higher among cases compared to cases in the preceding month; < 12 months pre-diagnosis | 4 months (increase in transvaginal ultrasound use) |
| **Gynaecological combined** | | | |
| Ewing (2016) | Sweden: primary care; cancers diagnosed 2011 and with 1+ primary care contact < 12 months pre-diagnosis; adults (N=327) | Visual identification (case-control); earliest week (reported as days) when rates among cases appeared to increase; < 12 months pre-diagnosis | 1-2 months (50 days) (increase in primary care consultations for any reason, increase in records of diagnostic codes in primary care) |
| **Breast** | | | |
| Morris (2017) | West Midlands, England: primary care; cancers diagnosed 1989-2006; adults 50-70 years (N=786) | Visual identification (case-only); earliest month when rates among cases appeared to increase; < 18 months pre-diagnosis | 3 months (increase in primary care consultations for breast-related symptoms) |
| Jensen (2018) | Denmark: primary care; cancers diagnosed 2009-2013; adults aged 50-90 years (N=18,396) | Statistical identification (case-only); earliest month when rates were significantly higher among cases compared to cases in the preceding month; < 18 months pre-diagnosis | 3 months (increase in primary care consultations for any reason) |
| Ewing (2016) | Sweden: primary care; cancers diagnosed 2011 and with 1+ primary care contact < 12 months pre-diagnosis; adults (N=947) | Visual identification (case-control); earliest week (reported as days) when rates among cases appeared to increase; < 12 months pre-diagnosis | 1-2 months (50 days) (increase in primary care consultations for any reason, increase in records of diagnostic codes in primary care) |
| Morrell (2019) | New South Wales, Australia: primary and secondary care; cancers diagnosed 2006-2015; adults aged 45 years + (N=1,999) | Statistical identification (case-control); earliest month when proportions were significantly higher among cases than controls; < 12 months pre-diagnosis** | 1 month (increase in GP consultations for any reason)** 1 month (increase in specialist consultations for any reason)** |
| Pottegard (2017) | Denmark: primary care; cancers diagnosed 2000-2012; adults (N=51,774) | Visual identification (case-control); earliest month when rates among cases appeared to increase; < 24 months pre-diagnosis | No increase pre-diagnosis (new first-time prescriptions use for any drug) |
| **Malignant melanoma** | | | |
| Ahrensberg (2016) | Denmark: primary care; cancers diagnosed 2002-2011; young adults 15-39 years (N=2,501) | Statistical identification (case-control); earliest month when rates were significantly higher among cases than controls (and increase was sustained); < 24 months pre-diagnosis** | 3 months (increase in primary care consultations for any reason) 2 months (increase in primary care blood test use) |
| Morrell (2019) | New South Wales, Australia: primary and secondary care; cancers diagnosed 2006-2015; adults aged 45 years + (N=2,070) | Statistical identification (case-control); earliest month when proportions were significantly higher among cases than controls; < 12 months pre-diagnosis** | 3 months (increase in GP consultations for any reason)** 2 months (increase in specialist consultations for any reason)** |
| Ewing (2016) | Sweden: primary care; cancers diagnosed 2011 and with 1+ primary care contact < 12 months pre-diagnosis; adults (N=459) | Visual identification (case-control); earliest week (reported as days) when rates among cases appeared to increase; < 12 months pre-diagnosis | 1-2 months (60 days) (increase in primary care consultations for any reason, increase in records of diagnostic codes in primary care) |
| Wang (2014) | UK; primary care; cancers diagnosed 1997-2006; adults (N=4,352) | Statistical identification (case-only); earliest month when rates were significantly higher among cases compared to cases in the preceding month (identified by literature review authors); < 24 months pre-diagnosis | 1 month (increase in primary care consultations for any reason)** |
| *The earliest point in time before diagnosis when a change was observed in a relevant clinical event type. Where multiple figures were given by a study for an event type or patient groups, the earliest single figure is shown. Therefore, the figure shown may only apply to specific groups of patients with that cancer. For studies using longer/ shorter time intervals than months (e.g. quarters, days), the equivalent range of months are highlighted. **Estimated by literature review authors using graphs or tables provided. ***Study included two different methods yielding different results; the results of primary focus in the study abstract/ conclusions are shown here. ****Sample size was not given for the specific cancer site. | | | |
